# Rule Modification’s Effects on the Feedback Type Given by Coaches at Young Football Levels

**DOI:** 10.3390/sports13030063

**Published:** 2025-02-20

**Authors:** Lidia Martinez-Jiménez, Ricardo André Birrento-Aguiar, Verónica Marco-Cramer, Enrique Ortega-Toro

**Affiliations:** 1 Faculty of Sport Sciences, University of Murcia, 30720 Santiago de la Ribera, Spain; lidia.martinezj@um.es (L.M.-J.); ra.birrentoaguiar@um.es (R.A.B.-A.); v.marcocramer@um.es (V.M.-C.); 2 Human Movement and Sports Sciences, Faculty of Sport Sciences, University of Murcia, 30720 Santiago de la Ribera, Spain; 3 Sports Performance Analysis Association, 30720 Santiago de la Ribera, Spain

**Keywords:** sport initiation, competition, under-10

## Abstract

Background: An adapted competition should create a favourable environment to tailor the feedback provided to the needs of young athletes. The aim of this study was to analyse the influence of rule modification on the type of feedback given by coaches to young football players. Method: The study sample consisted of four under-10 male coaches from four Spanish teams. The analysis was conducted using a quasi-experimental A-B design, in which two tournaments were played: Tournament 1 with the official Spanish Football Federation (RFEF) rules and Tournament 2 with rule modifications. All the feedback provided by the group of coaches during the two tournaments was recorded, yielding a total of 4.386 for Tournament 1 and 3.728 for Tournament 2. Results: The results showed that in both tournaments, the predominant feedback from the coaches was individual, prescriptive, affective, and non-valuable. However, they indicated that the orientation of the feedback and its autonomy positively varied during the adapted competition. The data obtained align with the results of other studies on the type of feedback given by coaches, despite some differing from scientific recommendations. Conclusions: It can be concluded that the modified rule competition promoted changes in the type of feedback provided by the coaches.

## 1. Introduction

Competition is a key component of the teaching–learning process in initiation sports, as it provides a practice that covers most of the learning situations that are essential for the training of players. At the same time, it offers massive practice if the player is active for a long time, which is the most beneficial for the training process [1,2]. Furthermore, competition promotes the transfer of learning [3,4] as it involves the construction and search for solutions to problems. Likewise, competition increases the motivation of participants and makes the teaching–learning process more attractive [5] and motivating [6] due to people’s ambition to compare themselves with others. Therefore, many authors support its use in the training of athletes [7,8].

Nevertheless, the current form of competition in youth sport is not conducive to achieving the goals and formative values that should be attained in the initiation phase [9,10]. In this sense, the scientific literature argues that for competition to be truly formative and meaningful, it must be adapted to the characteristics and level of the players [4,11,12], which would also increase the development of participants’ skills [13]. Various measures have been proposed to achieve the necessary adaptation, such as changing the rules or influencing the role of the coach [14]. In the case of badminton, it has been demonstrated that reducing the dimensions of the court and lowering the height of the net has a beneficial impact on the acquisition of skills among participants, encouraging the use of a greater variety of strokes [15]. In tennis, a reduction in racket size and weight, coupled with the use of larger and slower balls, has been shown to result in more accurate strokes by players [13]. Modifying the height of the net has been shown to encourage a more varied and assertive style of play, while also enhancing the motivation of the participants [16]. The alteration of the rules engenders a favourable experience for players, thereby enhancing their perception of self-efficacy [17].

Conversely, in team sports, alterations to the dimensions of the field in association football during training stages have been shown to encourage decision-making and facilitate the technical and tactical development of players [18]. The technical–tactical variability of young players in football is enhanced when aspects of the competition are modified, including the system of confrontations, the form of scoring and/or the size of the goal [19]. In beach handball, the adaptation of certain rules for trainee players up to the age of 11 and the modification of field dimensions and ball size, among other factors, has been shown to encourage player participation and adherence to the game [20]. Modifying the dimensions of the playing field to accommodate younger players, such as bringing the three-point line closer in basketball, results in alterations to the game conditions as players adapt their throwing movements, which in turn leads to a reduction in unfavourable movement patterns [21,22]. The actions of the coach have a significant impact on the learning outcomes of the players [23,24]. Nevertheless, coaches do not always conduct themselves in an appropriate manner, and they frequently exhibit negative or non-formative behaviours regarding values [25,26]. It is imperative that positive behaviours are the dominant force within the teaching–learning processes of beginner players [25]. Therefore, it is of the utmost importance that coaches receive comprehensive training and possess a clear understanding of the appropriate times and methods for providing feedback, in order to fully capitalise on its advantages [24].

Feedback can be defined as the set of information provided to an athlete or student after performing a motor execution, with the objective of influencing the same or the next action, thereby ensuring the acquisition of quality learning [27]. This process is aimed at closing the gap between current and desired learning outcomes [28]. The feedback, whether internal or external, that occurs between one action and another and that allows for modification of the execution or confirmation of the expected action is the determining factor in whether learning is meaningful [27]. Furthermore, it has been demonstrated that providing students with feedback can enhance their motivation and autonomy while also improving the classroom or team climate [29,30].

The literature offers recommendations for providing effective feedback, which are based on aspects related to the type of feedback, the timing of feedback, and the frequency of feedback [27,29]. The type of feedback can be classified in the following manner, as evidenced [22,27,31]: (a) direction (individual/group), indicating whether the information is addressed to a specific person or to several people; (b) emotional orientation (positive/negative/neutral), depending on the form and content of the feedback; and the intention of the feedback can be evaluative, descriptive, prescriptive, affective, or interrogative. Furthermore, some authors [32] have supplemented the classification of feedback types with the variable autonomy. The degree to which the type of feedback provided allows the individual to exert control over their teaching–learning process [33] has been evaluated [33].

A review of the scientific literature on the type of feedback that should be provided in competitive settings, with a particular focus on the initiation of sports, suggests a preference for the use of positive feedback [24,25]. In alignment with the intended outcome, feedback should be prescriptive, affective, and, on occasion, interrogative. The latter is recommended for guiding discovery learning [34,35]. In addition, coaches should provide feedback that is tailored to the individual [36]. Moreover, research indicates that appropriate feedback should facilitate the autonomy of the performer, thereby enhancing their experience of practice [37]. Additionally, feedback should convey values, as this is a primary objective in sport [38].

Sport constitutes an appropriate vehicle for the transmission of personal and social values to those who engage in it [39]. This is particularly salient in the context of sport initiation, where players are undergoing a process of maturational development at all levels, psychological, moral, and social [40]. These values can be further subdivided into those specifically associated with the practice of sport. For example, respect can be linked to fair play, sportsmanship, and tolerance [41,42]. While sport does not inherently possess educational value, the integration of values must be conducted through coaches, who should explicitly and concretely demonstrate the values they wish to in their athletes, and who should also utilise feedback in a manner like that employed with technical and tactical content [43]. These values are not currently evident in the competitions that take place, and the inherent competitiveness of such events has been found to result in a decrease in the inculcation of values [9]. In particular, the evidence suggests that younger football coaches are more likely to engage in negative behaviours toward their players, including the use of inappropriate language and expressions of regret, than positive behaviours [26].

The type of feedback employed by coaches varies depending on the stage of the match. Prescriptive feedback is more frequently used during the first half, whereas affective feedback is more prevalent during the second half [35]. This finding differs from another study, which found that affective feedback was the least frequently provided [27]. From an alternative perspective, some studies have indicated that most of the feedback provided by coaches is positive [44]. Regarding the direction of feedback, the literature does not indicate a clear preference for a specific type. On the one hand, coaches tend to provide group feedback more frequently [45]. On the other hand, during competitive play, they rely on individual feedback to a greater extent. The feedback provided by coaches is predominantly of a controlling nature that does not support player autonomy. Furthermore, the feedback they provide does not espouse values and does not refer to evaluative terms [32]. In alignment with the autonomy and values of feedback, it is noteworthy that many studies conducted to date have concentrated their data collection on questionnaires of coaches’ perceptions, thereby failing to analyse the feedback provided by coaches.

It can be reasonably deduced that a competition designed with the specific requirements of young athletes in mind would facilitate a more conducive setting for more effective feedback. The aim of this study was to investigate the effect of a competition designed to meet the needs of young players on the type of feedback provided by coaches in the context of youth football.

## 2. Materials and Methods

### 2.1. Participants

The study sample consisted of four football coaches in four Spanish teams of boys under 10. All the coaches were male, with a mean age of 31.45 ± 11.32 and a football coaching experience of 9.32 ± 7.23 years. All four coaches had a level II federative sports qualification.

### 2.2. Procedure

All feedback was recorded by the coaches during two tournaments (RFEF tournament = 4386; modified tournament = 3728) involving four teams, all playing against each other. In each match, both coaches were recorded; therefore, a total of 12 matches and 24 recordings were obtained. The study followed a quasi-experimental A-B design to analyse the effects of rule modification on different types of feedback. The independent variable of the study was the rules of the game. Two tournaments were played; the first one (RFEF tournament) followed the official RFEF regulations, and in the second one (modified tournament), the following modifications were made:

- Fair play’ scoring: Each team could score up to two points for “fair play”. One point was the average of the rating of the players themselves, the opponents, and the parents/guardians on a scale of 0 to 1. The other point was obtained by receiving a white card from the referee (positive attitudes) or, on the contrary, by subtracting that point (negative attitudes).
- Scoring for the competition: There were 3 points for a won match, 2 points for a drawn match and 1 point for a lost match. To this final score, the ‘fair play’ score mentioned above was added.
- Playing time: It consisted of five 10 min periods, with a 10 min break between the third and fourth periods.
- Scoreboard: The total score of the match was the sum of the periods won by each team.
- Goalkeeper: The goalkeeper had to play at least one period as a field player.
- Headers: Headers were not allowed, unless the ball had previously bounced.
- Goal kicks: Opposing players could only cross the offside line once the ball had been put into play.

The dependent variable was the feedback provided by the coaches. The feedback was classified following an ad hoc recording sheet previously validated by a panel of experts to ensure the validity of the content and wording [46]. Specifically, the type of feedback was analysed according to the following variables (Table 1).

The feedback data collection instrument was a microphone with a recording device (Sony ICD-PX240 4 Go, Tokyo, Japan). The microphone was placed on the coach’s chest at the start of each game and removed when the referee declared the game over. In this way, the information was recorded and facilitated subsequent analysis.

Four observers (graduates in physical activity and sports science) were trained to record the categories and types of feedback. Following observer training, inter-observer and intra-observer reliability was calculated. Prior to the analysis of the feedback, the observers were trained. After the training, inter-observer and intra-observer reliability tests were performed. To analyse reliability, 5 min of feedback was analysed, reporting minimum values of 0.95 for the intention variable (Table 2).

### 2.3. Statistical Analysis

Data analysis included descriptive analysis using absolute and relative frequency analysis. Then, to analyse possible relationships between the different categories of feedback and the different tournaments, an analysis was carried out using the chi-square test (*p* < 0.05). The Phi test (Phi) or Cramer’s V test (V) were used to calculate the effect size. Statistical analyses were performed using SPSS, v. 28.0 for Mac; SPSS Inc., Chicago, IL, USA.

## 3. Results

The results show that the total amount of feedback given by coaches during the regular tournament (n = 4386) is higher than the amount of feedback given during the modified tournament (n = 3728).

Table 3 shows the amount and percentage of feedback given by coaches by direction, distinguishing between feedback given during the regular tournament and feedback given during the modified tournament.

The data in Table 3 reflect a statistically significant relationship between the type of feedback direction and the type of tournament (ꭕ2 (1, n = 8114) = 6860, *p* < 0.05, effect size, *p* < 0.05 Phi = 0.029), with a lower percentage of individual feedback and a higher percentage of group feedback observed in the modified tournament. Thus, it can be observed that in both tournaments, almost all feedback was of an individual nature.

Table 4 shows the number and percentage of feedback types given according to their intention. In addition, a comparison of each type of feedback can be observed depending on whether it occurs in the RFEF tournament or in the modified tournament.

The results in Table 4 show statistically significant relationships between the variable feedback intention and the type of tournament (ꭕ2 (5, n = 8113) = 233.620, *p* < 0.05; effect size, *p* < 0.05, V = 0.170), such that in the modified tournament, a statistically higher percentage of descriptive and affective feedback was observed, and lower percentages of interrogative, prescriptive, explanatory, and evaluative feedback were observed. These data also show that more than half of the feedback given by the coaches in both the RFEF and the modified tournament was of the prescriptive type and more than a quarter of the affective type, leaving the remaining four types of feedback with very low percentages.

Table 5 details the amount and percentage of the types of feedback given by coaches based on emotional orientation, distinguishing between the RFEF tournament and the modified tournament.

The results showed statistically significant relationships between the variable feedback orientation and the type of tournament (χ2 (2, n = 8113) = 26.354, *p* < 0.05; effect size, *p* < 0.05, V = 0.057). In the modified tournament, there was a higher percentage of positive and neutral feedback and a lower percentage of negative feedback. Table 6 shows that in both tournaments, approximately half of the feedback was negative, and the other half was evenly split between neutral and positive feedback. However, it is worth noting that in Tournament 2, where the rules are changed, the percentage of negative feedback decreased, while the positive feedback increased. Table 6 shows the number and percentage of feedback used by the coaches according to the degree of information autonomy, differentiating between the normal and the modified tournament.

The data in Table 6 reflect statistically significant relationships between the variable feedback autonomy and the type of tournament (χ2 (4, n = 8113) = 73.609, *p* < 0.05; effect size, *p* < 0.05, V = 0.095), so that in the modified tournament, a lower percentage of controlling and neutral feedback and a higher percentage of supportive feedback were observed. Thus, it can be observed that in both tournaments, coaches used controlling feedback three out of four times. Nevertheless, feedback tended to change when the rules were changed, with a minimal decrease in controlling feedback and an increase in supportive feedback. Table 7 details the amount and percentage of feedback given based on whether it provided value, comparing the results from the normal tournament with the modified tournament.

The last set of data collected established a statistically significant relationship between the variable Feedback Values and the type of tournament (ꭕ2 (2, n = 8114) = 10.926, *p* < 0.05; effect size, *p* < 0.05, Phi = 0.037), with a higher percentage of feedback providing values and a lower percentage of feedback not providing values in the modified tournament. Thus, the results reflect that almost all the feedback given by the coaches was non-valuative, although this contribution is considered in the result because, despite the fact that the amount of value-giving feedback increased in the modified tournament, the percentage was still minimal.

## 4. Discussion

An adequate teaching–learning process includes a competition adapted to the needs of the participants and feedback based on scientific recommendations. Therefore, the aim of this study was to analyse how a competition adapted to the needs of young players influences the type of feedback given by youth football coaches.

In general, the results showed that the modification of rules, such as playing time, scoreboard, competition score, and fair play score, among others, slightly changed the type of feedback given by coaches, giving feedback that is closer to the recommendations of the scientific literature. In general, it was found that coaches gave more feedback in the normal tournament than in the modified tournament.

Depending on the direction of the feedback, it was found that most of the feedback given in both the normal and the modified tournament was of an individual nature, although in the modified tournament, the percentage of group feedback increased minimally, while individual feedback decreased. In addition, this greater use of individual feedback is in line with scientific recommendations regarding the orientations that feedback should have, depending on the direction, to positively influence the teaching–learning processes of young people [27,36].

From the perspective of feedback intentions, the results reflected that the change in rules between one tournament and another did not promote major changes in the type of feedback given by coaches. It was found that almost all feedback was prescriptive and affective feedback, both in the normal tournament and in the modified tournament. In the modified tournament, the use of modified feedback increased, while interrogative feedback remained similar and evaluative feedback decreased slightly. The data from this study are in line with the recommendations of the scientific literature on the type of feedback coaches should give based on intention, and with the results obtained by other authors in previous studies, since prescriptive and affective feedback predominate [35,44]. Nevertheless, the data differ from the results obtained in other studies, where affective feedback was the least used [24]. Regarding the emotional orientation of the feedback, it was found that the type of feedback changed positively when the rules were adapted, i.e., in the second modified rule. This is reflected in a percentage increase in positive and neutral feedback, while negative feedback decreased. This change from one tournament to another may have been due to the fact that the rule change encouraged coaches to reflect on their behaviour and what may not be appropriate, and more specifically, this change may have been influenced by the inclusion of the white card, which took into account the positive attitudes of coaches and players and could be counted in the competition scoreboard [41,46]. Although this change was positive, the feedback transmitted was still mostly negative, moving away from the guidelines that determine what constitutes appropriate feedback [24,25] and in line with the results of other studies analysing coaches’ feedback [47].

In line with feedback autonomy, the results showed that the percentage of supportive feedback increased in the modified tournament compared to the percentage in the normal tournament, while the opposite occurred with negative and neutral feedback, as their percentage decreased when the rules were modified. These results are positive because they reflect an influence of the rule change on the type of feedback given by the coach, which is more in line with what is considered appropriate feedback [37]. Nevertheless, it was also observed that the feedback based on autonomy that predominated in the coaches’ behaviour was the controlling type, with a high percentage. These data are consistent with other studies that have analysed the autonomy of feedback from coaches and found that a high percentage of coaches used controlling feedback [32], and the data differ from the recommendations of the scientific literature, which state that appropriate and beneficial feedback for students should support their autonomy [37].

Regarding the last variable analysed, values, it was found that almost all the feedback given by the coaches did not refer to evaluative terms, this being the case in both the normal and the modified tournament. Nevertheless, it was found that the percentage of feedback that provides values increases minimally during the modified tournament, which could indicate the beginning of a change due to the change in the rules and the introduction of the white card during the competition, as it is based on the recognition of positive attitudes and fair play, among other aspects [46]. The prevalence of feedback that does not provide values is consistent with the results obtained in other studies on what coaches do [22] and is completely different from what is determined by the scientific literature on what appropriate feedback should be based on with reference to evaluative terms [38,41]. Thus, the results obtained show that in the tournament in which a rule change was implemented and adapted to the characteristics of the participants, the type of feedback given by the coaches varied, although more markedly in some variables than in others. These changes bring the feedback closer to the keys established in the scientific literature to obtain the benefits of feedback, such as a higher-quality teaching–learning process, a better team climate, and an increase in the motivation and autonomy of the participants. The modification of the rules, especially those directly related to the training of educational values, can encourage coaches to reflect on their verbal behaviour, the need to adapt to players’ needs, and to consider other aspects that can benefit them in the competition, such as the white card.

Coaches are aware of the existence of this card and what they must do to get it, so they are more attentive to the type of feedback they give and try to modify it if necessary. All of this could be the reason for the results obtained and how the change in the rules influenced the type of feedback given by the coaches.

### Limitations

The main limitation is that the sample was only made up of boys due to the lack of female teams. Also, the influence of biological age on the coach’s feedback was not analysed. It would also be necessary to carry out studies with a wider range of teams to obtain more feedback from coaches, as well as analysing at different training levels.

## 5. Conclusions

The following can be concluded:

- The direction of feedback is mostly individual in both the normal and modified tournaments.
- The intention of the feedback, as well as the values it provides, does not vary across tournaments, as prescriptive, affective, non-valued feedback continues to predominate.
- The emotional orientation of the feedback changes when the fair play variable is introduced into the rules, decreasing negative feedback and increasing positive feedback.
- Coaches’ feedback is less controlling and more autonomy-supportive when the rules are modified.
- Changing the rules has a positive effect on the type of feedback given by coaches, encouraging the beginning of a change in this type of feedback and moving closer to the recommendations of the scientific literature on what constitutes appropriate feedback.
- These results invite the scientific community to analyse the feedback in different experimental studies with modified competitions in individual and team sports. In this way, it will be important to analyse the variables in the type of feedback in players with different levels of maturity development.

## Figures and Tables

**Table 1:** Study variables occurred in the record sheet.

Variable	Category	Description	Example
Direction	Single	Targeted at a specific player.	“You have gone that move well.”
	Group	Addressed to the group.	“We need to move the ball faster.”
Intention	Evaluative	Provides a qualitative estimation of the performance accompanied by a justification.	“The finish on goal was correct, because the defence left us space.”
	Descriptive	It involves the provision of information containing details about the execution of the movement or action performed.	“We unbalanced when you shot a goal.”
	Explanatory	Seeks to give empirical reasons for the execution error. It tells you the causes. It justifies the causes of the error. Biomechanical, psychological, etc., reasons are involved.	“When you hit the ball, your body was too far away from the ball, and you didn’t too far away from the ball, and you don’t have enough power.”
	Prescriptive	It provides information on how the next executions should be carried out.	“¡you have passed the ball in this situation!”
	Interrogative	It involves asking the player a question about the performance he/she has made, to make him/her reflect and become aware of his/her own performance.	“¿Where were your defence?”
	Emotional	Refers to information of an affective nature and is related to the emotional dimension of the player.	“Well done, Gonzalo, you are so good.”
Emotional orientation	Positive	Focuses on success.	“Good shooting at goal.”
	Negative	Focuses on the error.	“Don’t be selfish, pass the ball to your teammate.”
	Neutral	Focuses neither on the error nor on the effort.	
Autonomy	Support	Supports autonomy; empathises.	Coach’s questions, reflections…
	Neutral		
	Controller	Very direct instructions; does not encourage autonomy.	“Stop that dribble”
Values		If the coach alludes to values of respect or fair play.	“Don’t shot at the referee.”
Other type of feedback		Not contemplated in the classification.	Coach’s comments not directly addressed to the player (criticism, whispering, etc.).

**Table 2:** Training observer values.

Observation	Direction	Intention	Type	Autonomy	Values	Observers
Intra-Observation	1	0.956	0.832	0.896	1	A-A
Inter-Observation	1	0.906	0.909	0.844	1	A-B
	1	0.906	0.909	0.844	1	A-C
	1	1	1	0.844	1	A-D
	1	1	1	0.844	1	A-E

**Table 3:** Descriptive values of feedback—direction variables.

Feedback Direction	Tournament Type			
	Statistics	RFEF Tournament	Modified Tournament	Total
Single Feedback	Observed	4163	3488	7651
	Percentage	94.9%	93.6%	94.3%
Group Feedback	Observed	223	240	463
	Percentage	5.1%	6.4%	5.7%
Total *	Observed	4386	3728	8114

* Statistically significant *p* < 0.05. RFEF—Real Federación Española de Fútbol.

**Table 4:** Descriptive values of feedback—intention variables.

Feedback Intention	Tournament Type			
	Statistics	RFEF Tournament	Modified Tournament	Total
Evaluative feedback	Observed	187	124	311
	Percentage	4.3%	3.3%	3.8%
Descriptive feedback	Observed	96	242	338
	Percentage	2.2%	6.5%	4.2%
Explanatory feedback	Observed	158	0	158
	Percentage	3.6%	0.0%	1.9%
Prescriptive feedback	Observed	2633	2193	4826
	Percentage	60.0%	58.8%	59.5%
Interrogative feedback	Observed	129	91	220
	Percentage	2.9%	2.4%	2.7%
Affective feedback	Observed	1182	1078	2260
	Percentage	27.0%	28.9%	27.9%
Total *	Observed	4385	3728	8113

* Statistically significant *p* < 0.05. RFEF—Real Federación Española de Fútbol.

**Table 5:** Descriptive values of feedback—emotional orientation variables.

Feedback Emotional Orientation	Tournament Type			
	Statistics	RFEF Tournament	Modified Tournament	Total
Positive feedback	Observed	963	977	1940
	Percentage	22.0%	26.2%	23.9%
Negative feedback	Observed	2381	1831	4212
	Percentage	54.3%	49.1%	51.9%
Neutral feedback	Observed	1041	920	1961
	Percentage	23.7%	24.7%	24.2%
Total *	Observed	4385	3728	8113

* Statistically significant *p* < 0.05. RFEF—Real Federación Española de Fútbol.

**Table 6:** Descriptive values of autonomy feedback.

Autonomy Feedback	Tournament Type			
	Statistics	RFEF Tournament	Modified Tournament	Total
Support Feedback	Observed	790	891	1681
	Percentage	18.0%	23.9%	20.7%
Neutral Feedback	Observed	248	107	355
	Percentage	5.7%	2.9%	4.4%
Controller Feedback	Observed	3345	2730	6075
	Percentage	76.3%	73.2%	74.9%
Total *	Observed	4385	3728	8113

* Statistically significant *p* < 0.05. RFEF—Real Federación Española de Fútbol.

**Table 7:** Descriptive values of feedback.

Feedback Values	Tournament Type			
	Statistics	RFEF Tournament	Modified Tournament	Total
No values	Observed	4360	3681	8041
	Percentage	99.4%	98.7%	99.1%
Values	Observed	25	47	72
	Percentage	0.6%	1.3%	0.9%
Total *	Observed	4386	3728	8114

* Statistically significant *p* < 0.05. RFEF—Real Federación Española de Fútbol.

## Data Availability

Data is contained within the article.

## References

[1] Merino A., Arraiz-Pérez A., Sabirón-Sierra F. (2016). Procesos formativos en el fútbol prebenjamín: El paso de la ingenuidad a la institucionalización. Educ. Fís. Cienc..

[2] Ortega E. (2004). Análisis de la Participación del Jugador con Balón en Etapas de Formación en Baloncesto (14–16 años) y su Relación con la Autoeficacia. Ph.D. Thesis.

[3] Ruiz L.M. (1997). Deporte y Aprendizaje, Procesos de Adquisición y Desarrollo de Habilidades.

[4] Piñar M.I., Cárdenas D. (2010). La competición como herramienta formativa: Diferentes propuestas en minibasket. Rev. Wanceulen Ef. Digit..

[5] Jiménez G., Recio J.A., Díaz B., Flórez G. (2012). Uso de competiciones y sistemas de clasificación como metodología de evaluación de una asignatura. En Asociación de Enseñantes Universitarios de la Informática (Eds.). Jorn. Enseñanza Informática.

[6] Reverter J., Plaza D., Mayolas C., Adell L. (2009). La competición deportiva como medio de enseñanza en los centros educativos de primaria. Retos.

[7] Manzino C., Rodríguez V. (2021). Desigualdades en el acceso a la competición deportiva: El caso de las escuelas de Montevideo (Uruguay). Lect. Educac. Fís. Deporte.

[8] Sánchez P. (2008). Actividad física y deportiva en las diferentes edades. Innov. Exper. Educ..

[9] Buszard T., Farrow D., Reid M. (2020). Designing Junior Sport to Maximize Potential: The Knowns, Unknowns, and Paradoxes of Scaling Sport. Front. Psychol..

[10] Ticó J. (2009). La competición en el deporte de base: ¿educación o perversión?. Cuad. Psicol. Deporte.

[11] Correia V., Carvalho J., Araújo D., Pereira E., Davids K. (2019). Principles of nonlinear pedagogy in sport practice. Phys. Educ. Sport Pedagog..

[12] McCarthy J., Bergholz L., Bartlett M. (2016). Re-Designing Youth Sport: Change the Game.

[13] Buszard T., Garofolini A., Reid M., Farrow D., Oppici L., Whiteside D. (2020). Scaling sports equipment for children promotes functional movement variability. Sci. Rep..

[14] Ortega E., Piñar I., Salado J., Palao J.M., Gómez M.A. (2012). Opinión de expertos y entrenadores sobre el reglamento de la competición infantil en baloncesto. RICYDE.

[15] Blanca J.C. (2021). Incidencia de las Modificaciones Reglamentarias Sobre el Proceso de Aprendizaje en Badminton. Ph.D. Thesis.

[16] Timmerman E., De Water J., Kachel K., Reid M., Farrow D., Savelsbergh G. (2015). The effect of equipment scaling on children’s sport performance: The case for tennis. J. Sports Sci..

[17] Giménez-Egido J.M., Carvalho J., Araújo D., Ortega-Toro E. (2023). Perceived self-efficacy by Under-10 tennis players when scaling the equipment and play area. Psychol. Sport Exerc..

[18] Vilar L., Duarte R., Silva P., Chow J.Y., Davids K. (2014). The influence of pitch dimensions on performance during small-sided and conditioned soccer games. J. Sports Sci..

[19] García-Angulo A., Palao J.M., Giménez-Egido J.M., García-Angulo F.J., Ortega-Toro E. (2020). Effect of the Modification of the Number of Players, the Size of the Goal, and the Size of the Field in Competition on the Play Actions in U-12 Male Football. Int. J. Environ. Res. Public Health.

[20] Sánchez-Sáez J.A., Morillo-Baro J.P., Sánchez J.M., Lara D., Arias-Estero J.L. (2022). Estudio piloto sobre las respuestas motrices y psicológicas de jugadores y entrenadores durante la competición a la propuesta de reglas para minibalonmano playa. Retos.

[21] Thomas C., Nolte K., Schmidt M., Jaitner T. (2023). Lower Baskets and Smaller Balls Influence Mini-Basketball Players’ Throwing Motions. Biomechanics.

[22] Viciana J., Cervelló E., Ramírez J., San-Matías J., Requena B. (2003). Influencia del feedback positivo y negativo en alumnos de secundaria sobre el clima ego-tarea percibido, la valoración de la EF y la preferencia en la complejidad de las tareas de clase. Eur. J. Hum. Mov..

[23] Sánchez D.L., Viciana J. (2002). Análisis del discurso de un entrenador de fútbol: Comparación entre dos situaciones diferentes de competición. Eur. J. Hum. Mov..

[24] Ortín G. (2018). Use of Feedback and Motivation in the Players. Comparison Between an Expert Coach and a New Coach. Bachelor’s Thesis.

[25] Pinheiro V., Camerino O., Richheimer P. (2014). Fair play in sport initiation, a study of football coaches. Retos.

[26] Viciana J., Zabala M. (2004). El papel educativo y la responsabilidad de los entrenadores deportivos. Una investigación sobre las instrucciones a escolares en fútbol de competición. Rev. Educ..

[27] Mamani-Ramos Á.A. (2020). El Feedback Como Instrumento de Aprendizaje en Educación Física y Deporte: La Clave del Éxito.

[28] Stobart G. (2005). Fairnessin multicultural assessment systems. Assess. Educ..

[29] Hattie J., Clarke S. (2020). Aprendizaje Visible: Feedback.

[30] Huéscar H., Barrachina J., Moreno J.A. (2022). En Búsqueda de la Autonomía en Educación Física.

[31] Palao-Andrés J.M., Hernández-Hernández E., Guerrero-Cruz P., Ortega-Toro E. (2011). Efecto de distintas estrategias de presentación de feedback mediante vídeo en clases de Educación Física. Apunts.

[32] Martínez-Jiménez L., Ortega-Toro E. (2023). Estudio del feedback de entrenadores/as de voleibol a jugadoras durante la competición en etapa formativa. JUMP.

[33] García I., Bustos R.B. (2020). Desarrollo de la autonomía y la autorregulación en estudiantes universitarios: Una experiencia de investigación y mediación. Sinéctica.

[34] Fonseca-Morales A., Martínez-Gallego R. (2022). Feedback y aprendizaje en el tenis: Conceptualización, clasificación e implicaciones prácticas. ITF Coach Sport Sci. Rev..

[35] Sánchez-Oliva D., Sánchez-Miguel P.A., Amado-Alonso D., Marcos F., García-Calvo T. (2010). Análisis de la conducta verbal del entrenador de fútbol en función de su formación federativa y del periodo del partido en categorías inferiores. Retos.

[36] Boud D., Molloy E. (2013). Feedback in Higher and Professional Education: Understanding It and Doing It Well.

[37] Hernández V.M., Santana P.J., Sosa J.J. (2021). Feedback y autorregulación del aprendizaje en educación superior. Rev. Investig. Educ..

[38] Unesco (2019). Educación de Valores a Través del Deporte.

[39] Cecchini J.A., Montero J., Peña J.V. (2003). Repercusiones del programa de intervención para desarrollar la responsabilidad personal y social de Hellison sobre los comportamientos de fair-play y el auto-control. Psicothema.

[40] Weinberg R.S., Gould D. (2010). Fundam Psicol Deport Ejerc Fís.

[41] Ortega-Vila G., Alarcón-López F., Giménez Fuentes-Guerra F.J., Ortega-Toro E., Robles-Rodríguez J., Abad-Robles M.T. (2022). El modelo de iniciación al fútbol y educación en valores de la Fundación Real Madrid. JUMP.

[42] Sánchez-Alcaraz B.J., López G., Valero A., Gómez-Mármol A. (2017). Los programas de educación en valores a través de la educación física y el deporte. Act. Fís. Deport. Cienc. Prof..

[43] Fernández A. (2023). Transmitiendo Valores: El Papel de la Fundación del Real Madrid; Sesión de conferencia. In IV Jornadas de Transferencia: De la Ciencia a Los Entrenadores, Murcia, Spain.

[44] Carreras J.C., Fuentes-Gimenez F.J. (2010). Methodology of education used in the education of the tennis during the stage of initiation. Retos.

[45] Giannousi M., Mountaki F., Karamousalidis G., Bebetsos G., Kioumourtzoglou E. (2016). Coaching Behaviors and the Type of Feedback They Provide to Young Volleyball Athletes. J. Phys. Educ. Sport.

[46] Ortega-Vila G., Franco J., Giménez J., Durán J., Jiménez J., Jiménez P.J., Lambert J. (2016). An evaluation of the” white card” as a resource for promoting an educational sports competition. J. Hum. Sport Exerc..

[47] Pérez B.L., Seoane A.M., García M.S. (2015). Differences Between Perceived and Registered Behavior of Basketball Coaches After Shot. SAGE Open.

